# Supplementary figures and images for: Phorbolester-activated Munc13-1 and ubMunc13-2 exert opposing effects on dense-core vesicle secretion
Source: eLife. 2022 Oct 10;11:e79433. doi: 10.7554/eLife.79433 (PMC9581527; doi:10.7554/eLife.79433)

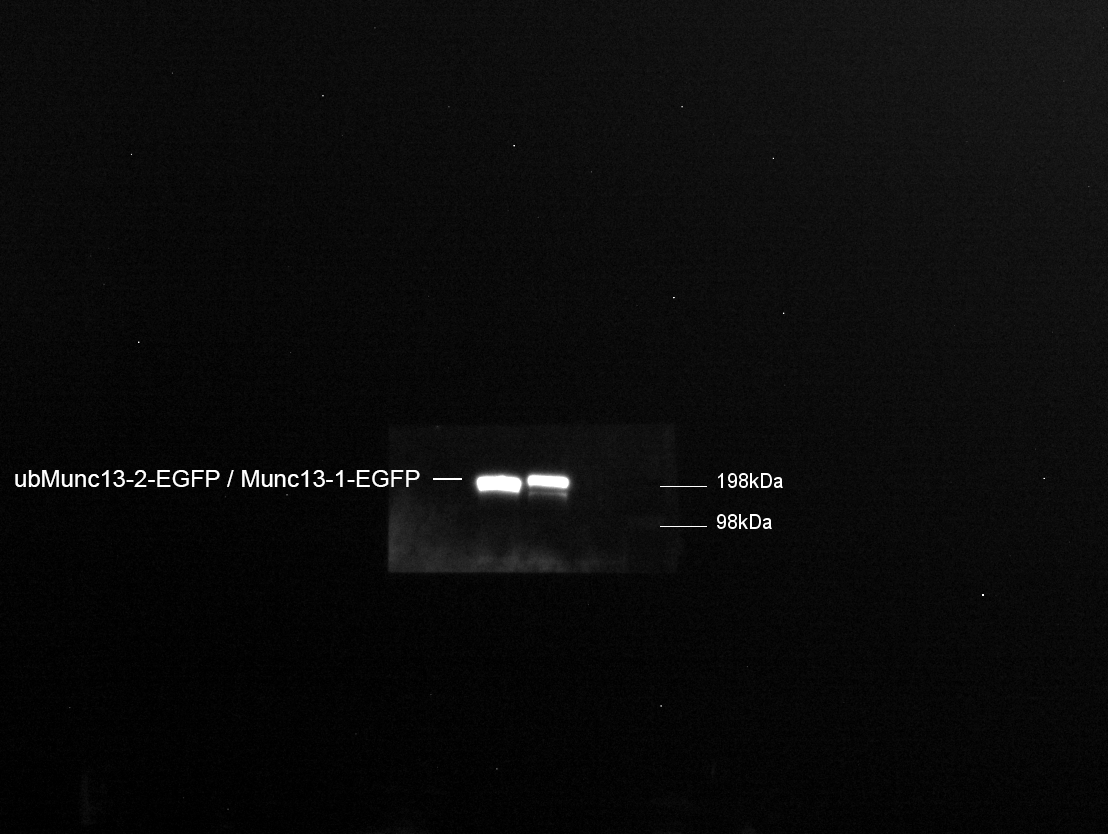

Supplement: Figure 3—figure supplement 3—source data 1. [file elife-79433-fig3-figsupp3-data1.zip › Figure 3 - Figure supplement 3 - source data/Figure3-Figure supplement 3-source data 1.tif]

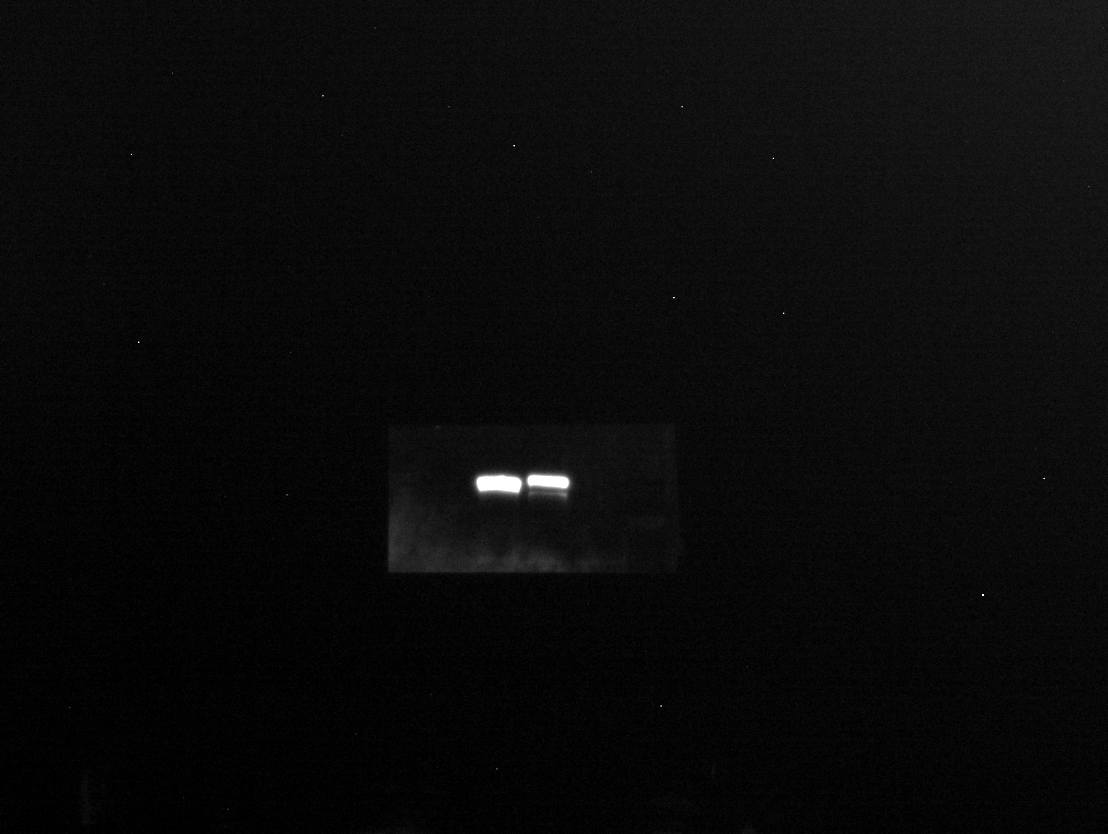

Supplement: Figure 3—figure supplement 3—source data 1. [file elife-79433-fig3-figsupp3-data1.zip › Figure 3 - Figure supplement 3 - source data/Figure3-Figure supplement 3-source data 2.tif]

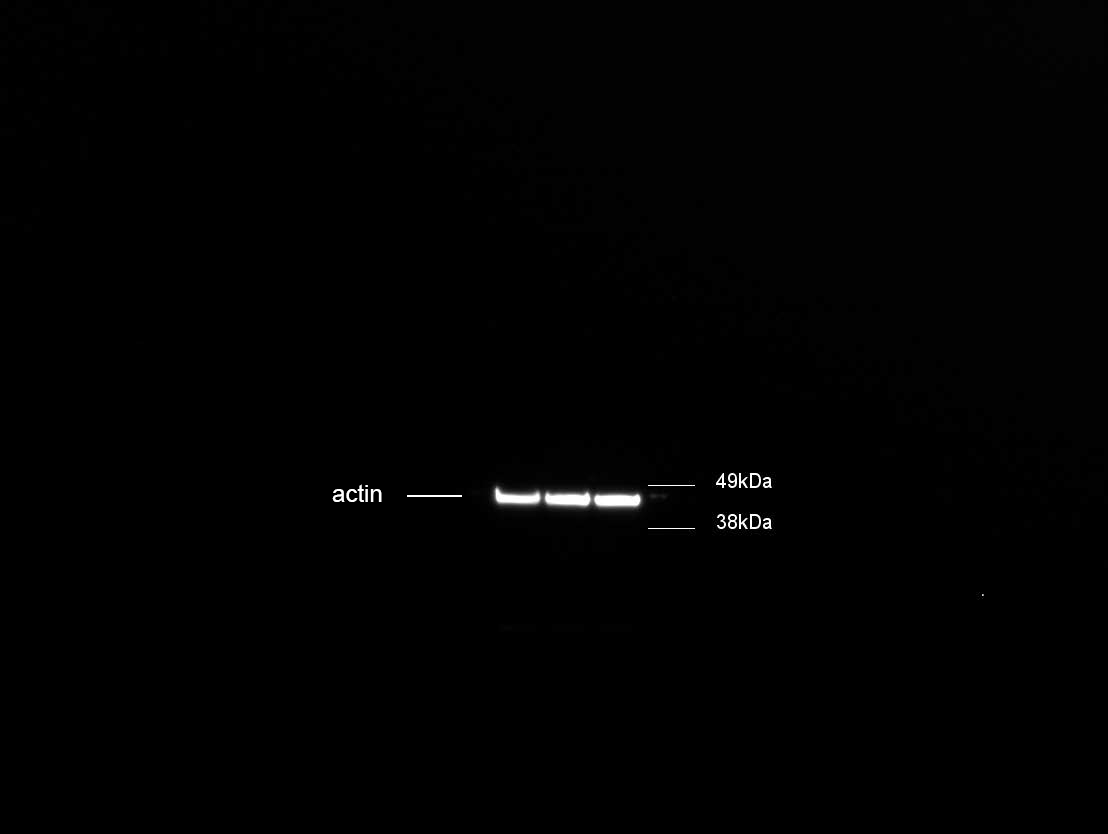

Supplement: Figure 3—figure supplement 3—source data 1. [file elife-79433-fig3-figsupp3-data1.zip › Figure 3 - Figure supplement 3 - source data/Figure3-Figure supplement 3-source data 3.tif]

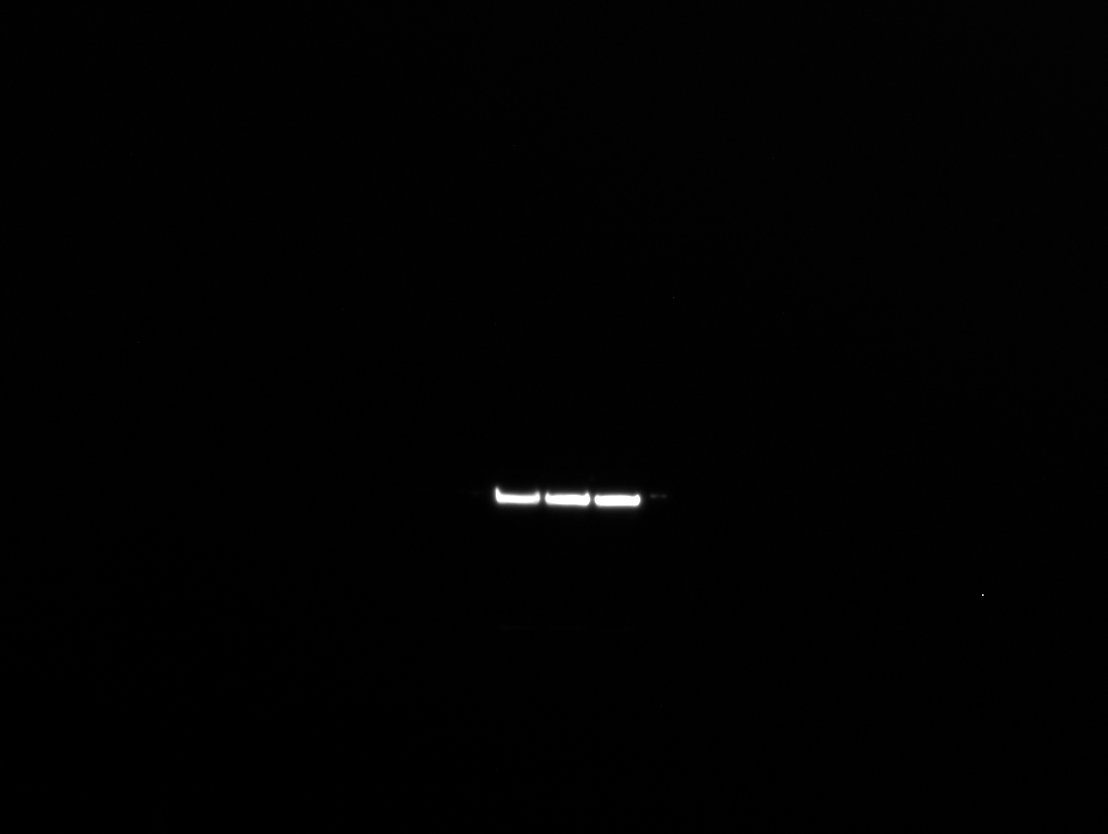

Supplement: Figure 3—figure supplement 3—source data 1. [file elife-79433-fig3-figsupp3-data1.zip › Figure 3 - Figure supplement 3 - source data/Figure3-Figure supplement 3-source data 4.tif]

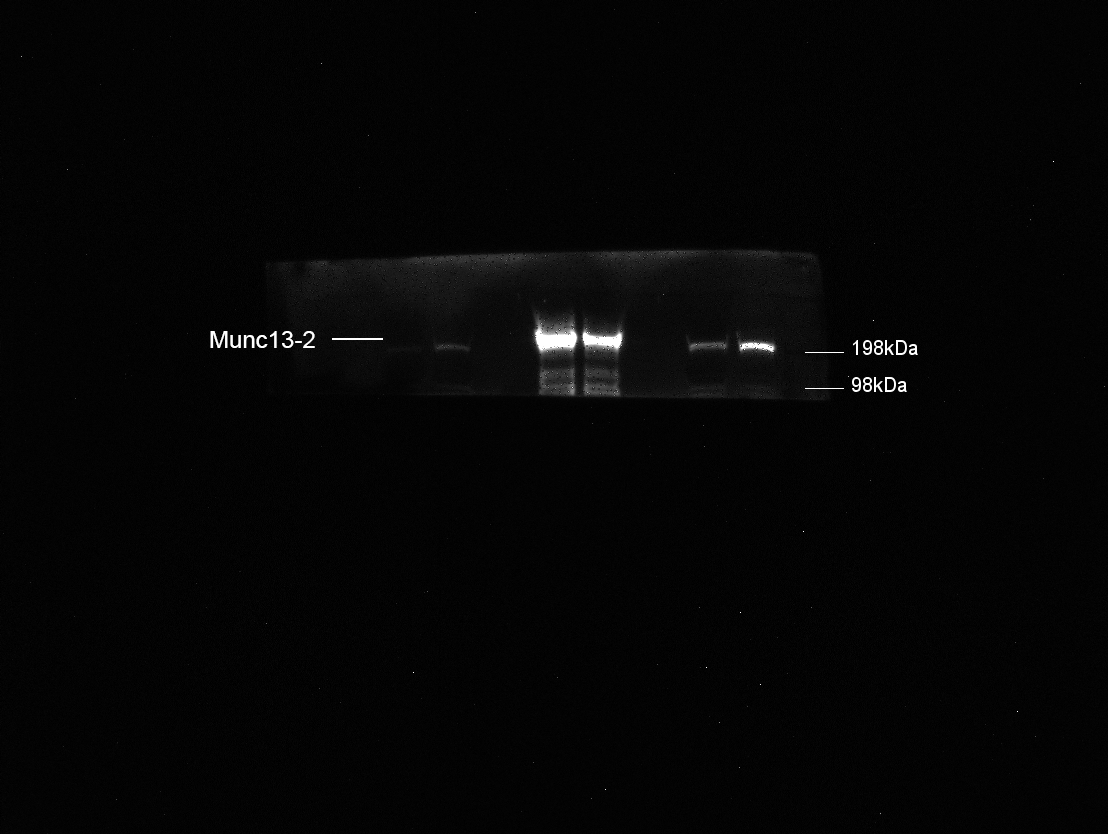

Supplement: Figure 6—figure supplement 1—source data 1. — Note that the file marked Figure 6-Figure supplement 1 - source data 6 contains two blots unrelated to the present manuscript. [file elife-79433-fig6-figsupp1-data1.zip › Figure 6 - Figure supplement 1 - source data/Figure 6-Figure supplement 1-source data 1.tif]

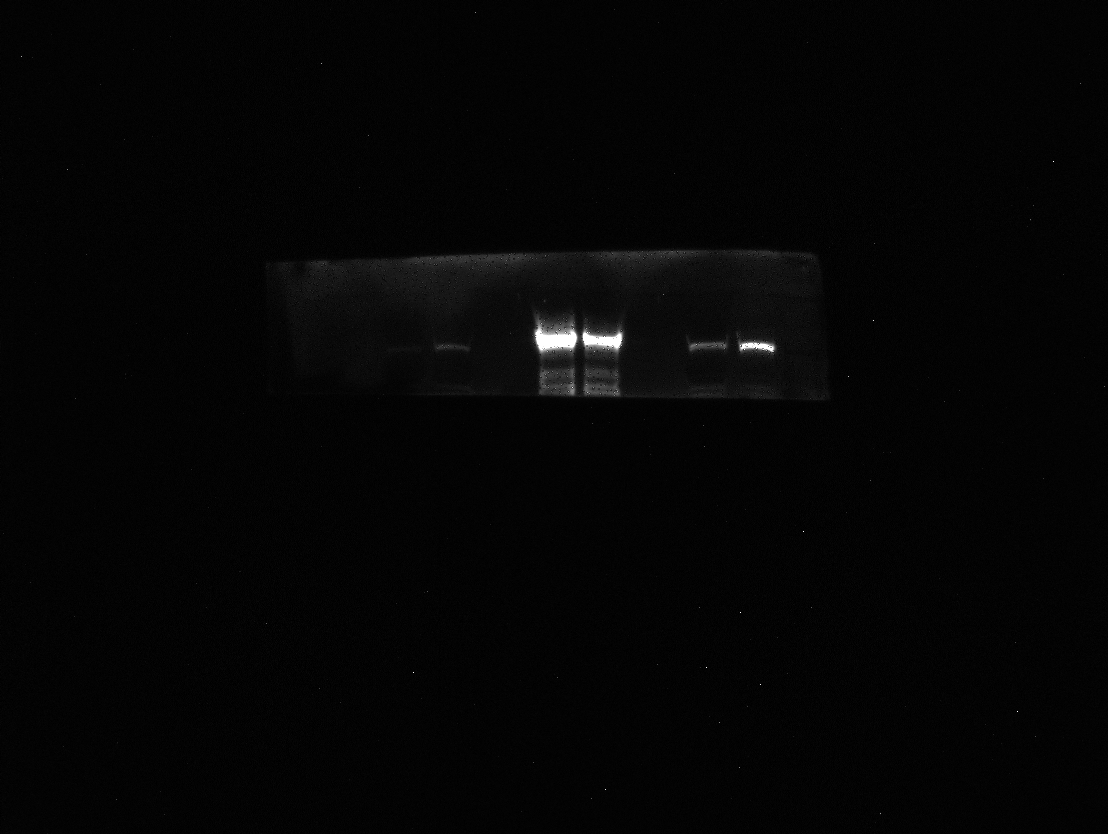

Supplement: Figure 6—figure supplement 1—source data 1. — Note that the file marked Figure 6-Figure supplement 1 - source data 6 contains two blots unrelated to the present manuscript. [file elife-79433-fig6-figsupp1-data1.zip › Figure 6 - Figure supplement 1 - source data/Figure 6-Figure supplement 1-source data 2.tif]

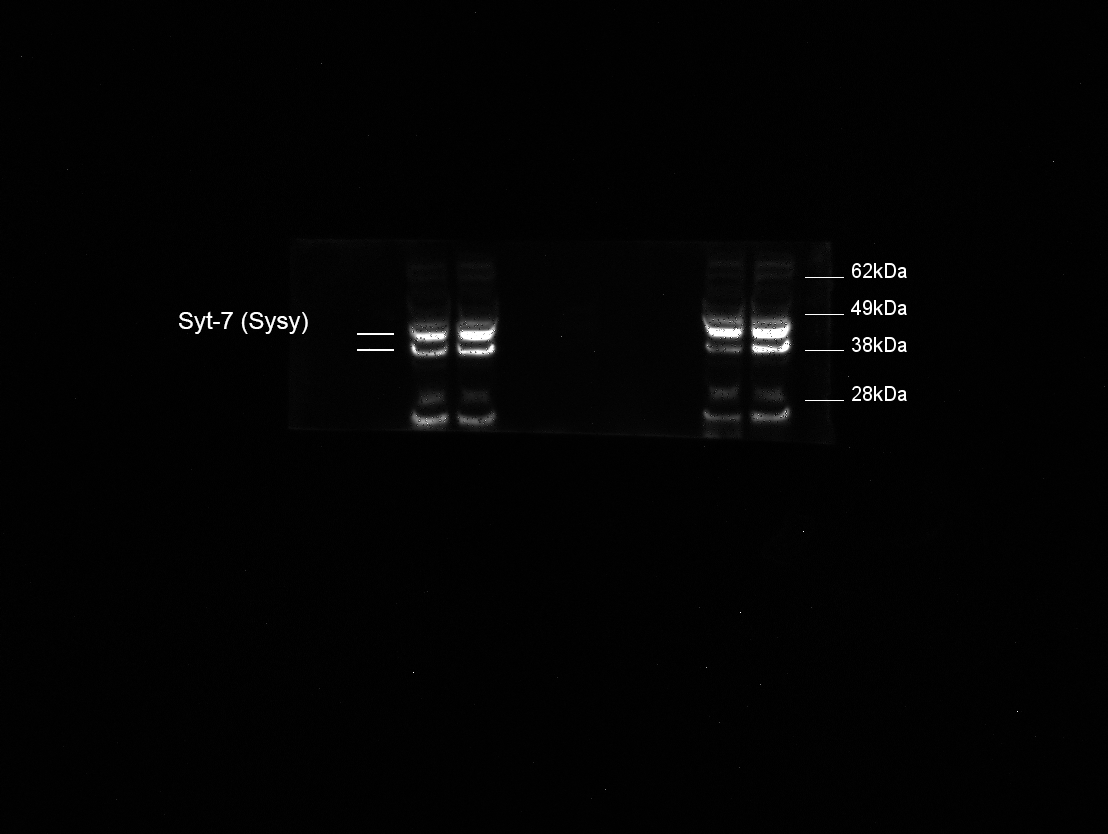

Supplement: Figure 6—figure supplement 1—source data 1. — Note that the file marked Figure 6-Figure supplement 1 - source data 6 contains two blots unrelated to the present manuscript. [file elife-79433-fig6-figsupp1-data1.zip › Figure 6 - Figure supplement 1 - source data/Figure 6-Figure supplement 1-source data 3.tif]

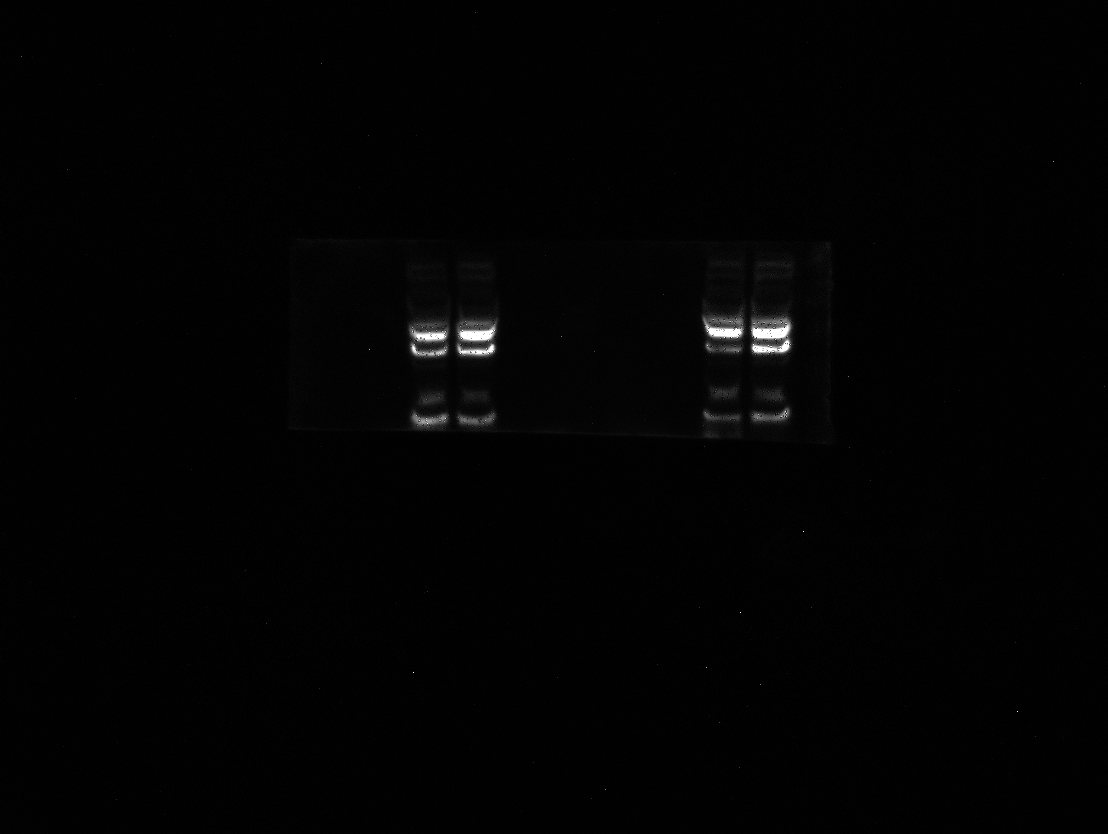

Supplement: Figure 6—figure supplement 1—source data 1. — Note that the file marked Figure 6-Figure supplement 1 - source data 6 contains two blots unrelated to the present manuscript. [file elife-79433-fig6-figsupp1-data1.zip › Figure 6 - Figure supplement 1 - source data/Figure 6-Figure supplement 1-source data 4.tif]

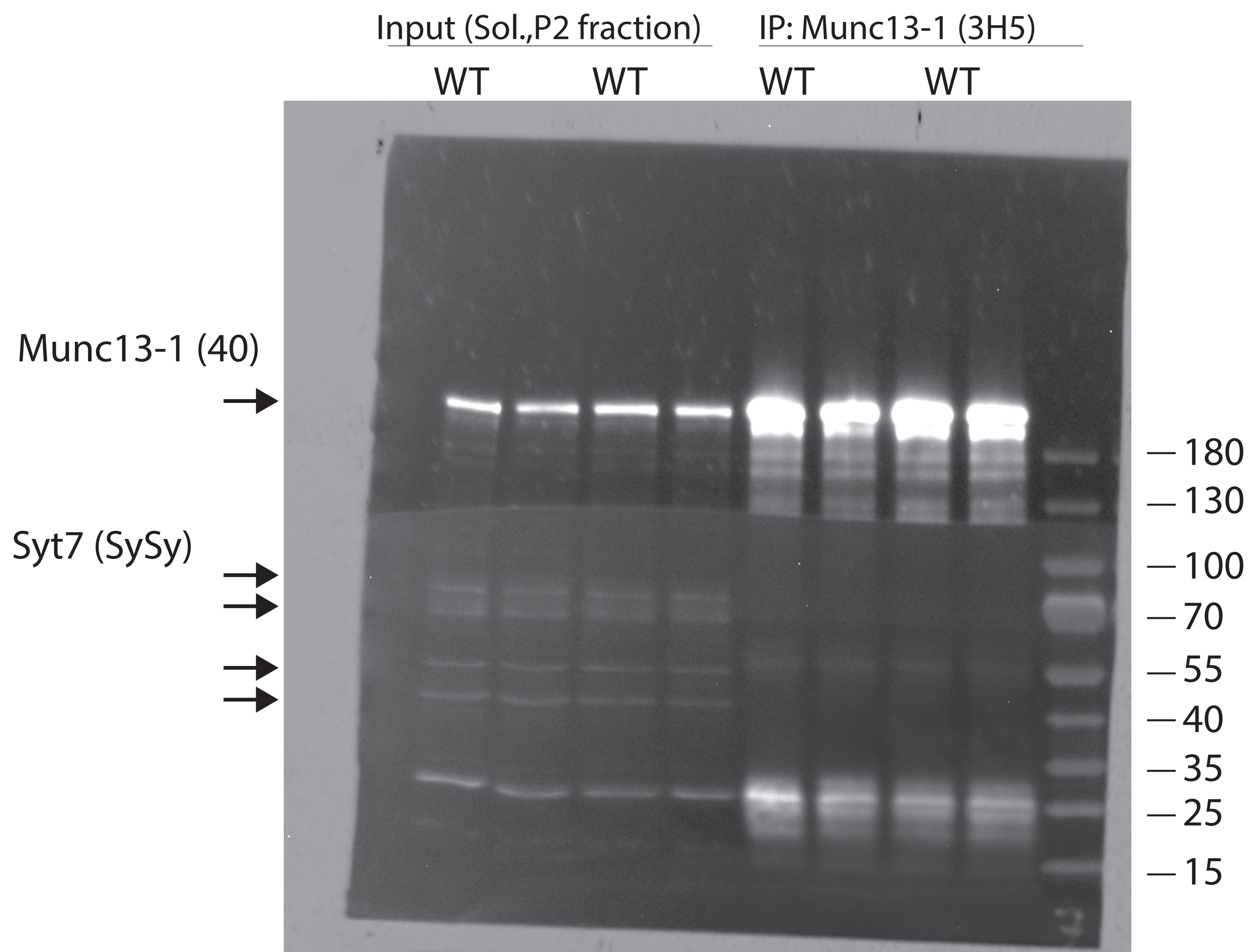

Supplement: Figure 6—figure supplement 1—source data 1. — Note that the file marked Figure 6-Figure supplement 1 - source data 6 contains two blots unrelated to the present manuscript. [file elife-79433-fig6-figsupp1-data1.zip › Figure 6 - Figure supplement 1 - source data/Figure 6-Figure supplement 1-source data 5.pdf]

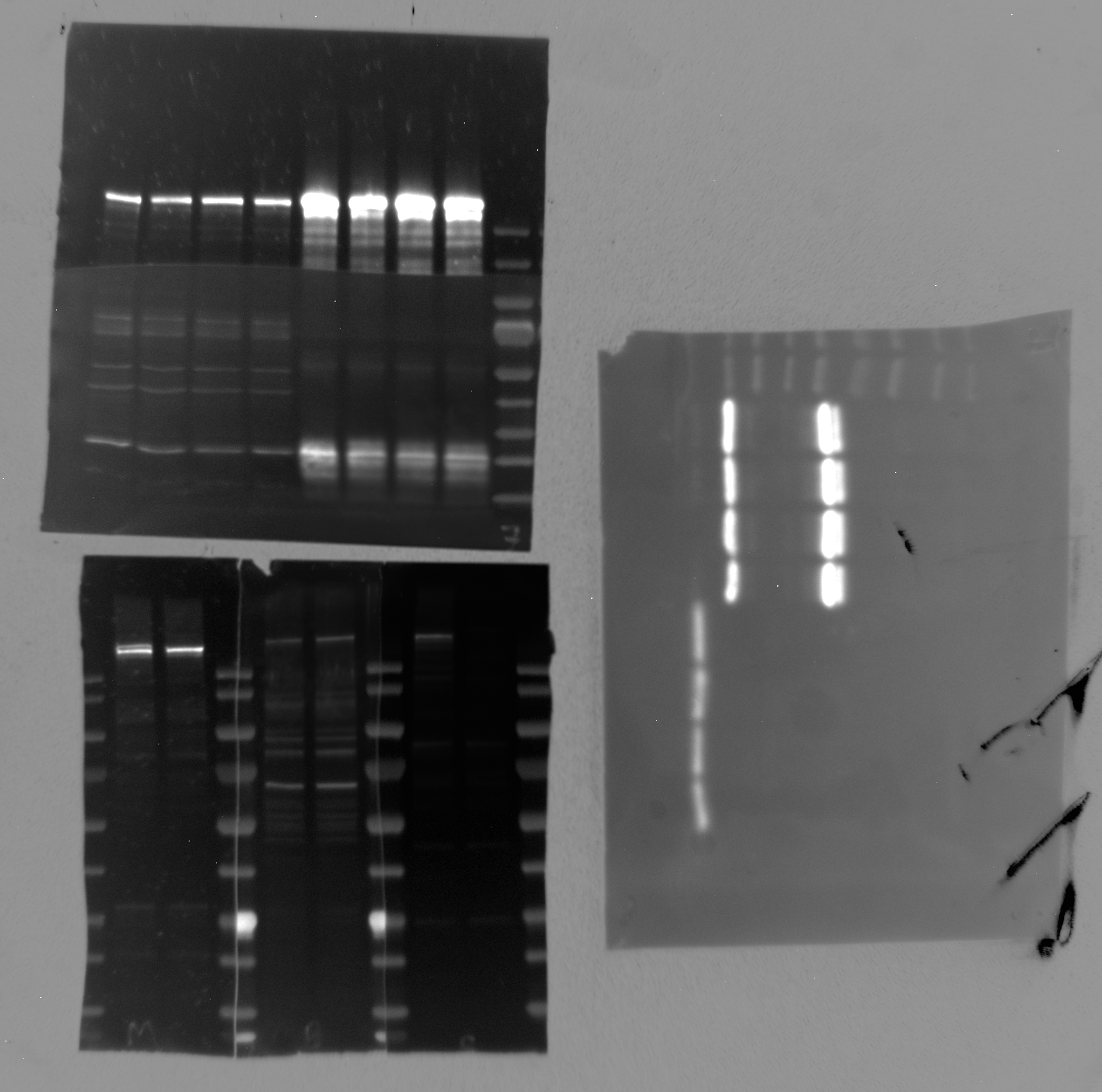

Supplement: Figure 6—figure supplement 1—source data 1. — Note that the file marked Figure 6-Figure supplement 1 - source data 6 contains two blots unrelated to the present manuscript. [file elife-79433-fig6-figsupp1-data1.zip › Figure 6 - Figure supplement 1 - source data/Figure 6-Figure supplement 1-source data 6.tif]
